# Do school-based prevention programs impact co-occurring alcohol use and psychological distress during adolescence?

**DOI:** 10.1017/S0033291724002897

**Published:** 2024-12

**Authors:** J. Halladay, M. Sunderland, N. C. Newton, S. J. Lynch, C. Chapman, L. Stapinski, J. L. Andrews, L. Birrell, M. Teesson, T. Slade

**Affiliations:** 1 School of Nursing, McMaster University, Hamilton, ON, Canada; 2 The Matilda Centre for Research in Mental Health and Substance Use, University of Sydney, Sydney, NSW, Australia; 3 Peter Boris Centre for Addictions Research, McMaster University/St. Joseph's Healthcare Hamilton, Hamilton, ON, Canada; 4 Faculty of Medicine, Department of Psychiatry and Addiction, University of Montreal, Montreal, QC, Canada; 5 Azrieli Research Center of the CHU Ste Justine Mother-Child University Hospital, Montréal, QC, Canada; 6 Department of Experimental Psychology, University of Oxford, Oxford, UK

**Keywords:** adolescent, alcohol drinking, prevention, psychological distress, student

## Abstract

**Background:**

Adolescence is a critical period for preventing substance use and mental health concerns, often targeted through separate school-based programs. However, co-occurrence is common and is related to worse outcomes. This study explores prevention effects of leading school-based prevention programs on co-occurring alcohol use and psychological distress.

**Methods:**

Data from two Australian cluster randomized trials involving 8576 students in 97 schools were harmonized for analysis. Students received either health education (control) or one of five prevention programs (e.g. Climate Schools, PreVenture) with assessments at baseline and 6, 12, 24, and 30 or 36 months (from ages ~13–16). Multilevel multinomial regressions were used to predict the relative risk ratios (RRs) of students reporting co-occurring early alcohol use and psychological distress, alcohol use only, distress only, or neither (reference) across programs.

**Results:**

The combined Climate Schools: Alcohol and Cannabis and Climate Schools: Mental Health courses (CSC) as well as the PreVenture program reduced the risk of adolescents reporting co-occurring alcohol use and psychological distress (36 months RR_CSC_ = 0.37; RR_PreVenture_ = 0.22). Other evaluated programs (excluding Climate Schools: Mental Health) only appeared effective for reducing the risk of alcohol use that occurred without distress.

**Conclusions:**

Evidence-based programs exist that reduce the risk of early alcohol use with and without co-occurring psychological distress, though preventing psychological distress alone requires further exploration. Prevention programs appear to have different effects depending on whether alcohol use and distress present on their own or together, thus suggesting the need for tailored prevention strategies.

## Introduction

Adolescence is the peak period of onset for lifetime mental health (MH) problems and substance use (SU) (Solmi et al., 2021), thus representing a critical time to prevent the incidence and chronicity of problems. Given adolescents spend much of their time in school, schools are well positioned to deliver prevention programs. Existing school-based programs tend to target SU or MH problems separately and evaluate them as distinct outcomes (Onrust, Otten, Lammers, & Smit, 2016; Werner-Seidler et al., 2021). However, the onset of co-occurring SU and MH problems is common among adolescents in school (Halladay, MacKillop, Munn, Amlung, & Georgiades, 2022) and co-occurrence is related to greater severity, complexity, and poorer treatment outcomes (Baker et al., 2007; Brière, Rohde, Seeley, Klein, & Lewinsohn, 2014; Wilkinson, Halpern, & Herring, 2016). Co-occurrence may occur due to MH problems leading to SU as a form of self-medication, SU leading to MH problems through substance-driven neurobiological or psychosocial changes, and/or shared common risk factors related to the development of both (Casey, Getz, & Galvan, 2008; Hussong, Jones, Stein, Baucom, & Boeding, 2011; Vanyukov & Ridenour, 2012). While co-occurrence is common and concerning, it is not universal. Adolescents can use substances without experiencing MH problems, and many adolescents with MH problems do not experience problems with substances (Halladay et al., 2022, 2020). As such, school-based prevention may benefit from taking a concurrent approach to prevention and evaluation that considers this complexity, rather than focusing solely on individual disorders and behaviors.

Alcohol use and emotional problems (depression, anxiety, distress, suicidality) are the most common SU and MH problems among adolescents (Australian Institute of Health and Welfare, 2020; Lawrence et al., 2015). These are key targets for prevention given harmful alcohol consumption is among the leading modifiable factors related to global disease burden (World Health Organization, 2018) and emotional problems contribute to the majority of MH-related hospital presentations that have been showing recent increases (Cutler et al., 2019). While the prevalence of alcohol use has declined among adolescents across high-income countries over the past two decades, emotional problems have shown steep increases (Halladay, Sunderland, Chapman, Teesson, & Slade, 2024). Despite diverging independent trends, most existing research characterizing trends in co-occurring problems show either consistency or strengthening of associations over time (Halladay et al., 2024). As such, risk and protective factors related to alcohol use and emotional problems may differ depending on whether or not they co-occur, as the experience of emotional problems may differ depending on whether substances are used (and vice-versa). Given existing prevention programs for alcohol use and emotional problems evaluate outcomes separately, it is unclear whether these programs are equally benefiting adolescents with and without co-occurring problems and how this may be impacting population trends.

Prevention in schools is typically addressed through multi-tiered systems of support, including: tier 1, *universal* interventions targeting all students, such as programs embedded into school curricula, and tier 2, *selective* interventions delivered to a sub-set of ‘at risk’ adolescents. The Climate and PreVenture trial (CAP; 2012–2015) was the first school-based SU prevention trial (focused on alcohol and cannabis) that combined universal and selective interventions (Newton, Teesson, Barrett, Slade, & Conrod, 2012). The Climate Schools Combined trial (CSC; 2014–2017) was the first combined school-based universal prevention trial for both MH (namely, depression and anxiety) and SU (namely, alcohol and cannabis) (Teesson et al., 2014). These trials represent world-first studies that explore effects of school-based prevention programs focused on alcohol use and emotional symptoms separately, and provide unique opportunities to explore their impacts on co-occurrence.

Climate Schools programs (recently rebranded as OurFutures, though content and delivery remain the same) are universal (tier 1), e-health, curriculum-based prevention programs for SU and MH problems (Teesson et al., 2014). The co-designed programs combine interactive online cartoon storyboards with manualized teacher-facilitated in-class activities. Each session includes a 20-min online storyboard with a 20-min in-person class discussion. The Climate Schools courses implemented as part of the CAP and CSC trials were the: (1) Climate Schools: Alcohol and Cannabis course (12 lessons over 6 months in year 8 [~age 13], hereby referred to as Climate SU) grounded in principles of harm-reduction and social-influence (Newton, Vogl, Teesson, & Andrews, 2011), and (2) Climate Schools: Mental Health course (6 lessons over 6 months in year 9 [~age 14], hereby referred to as Climate MH) grounded in cognitive behavioral therapy (CBT) with related psychoeducation and skills-training (Teesson et al., 2020).

The Climate SU course has demonstrated a variety of alcohol-related benefits (e.g. reduced odds of initiation and reductions in growth of alcohol use, binge drinking, and alcohol-related harms) when compared to standard health education across five Australian randomized trials involving over 11 000 students in 130 schools (Champion et al., 2016; Newton, Andrews, Teesson, & Vogl, 2009a, 2009b, 2010, 2022a, 2022c; Slade et al., 2021; Teesson et al., 2017, 2020, 2024; Vogl et al., 2009). Most outcomes are assessed up to 3 years from baseline, though two studies have shown alcohol-related benefits to be maintained for up to 6–7 years (Newton et al., 2022c; Teesson et al., 2024). On the other hand, evaluations of the Climate MH course have demonstrated mixed results. The original longer version of the course suggested reductions in depressive and anxiety symptoms immediately following the intervention, compared to control (Wong, Kady, Mewton, Sunderland, & Andrews, 2014). The six-session updated version, when combined with Climate SU, slowed growth in depressive and anxiety symptoms as well as reduced odds of any drinking and heavy episodic drinking up to 30 months post-intervention (Teesson et al., 2020); however, the MH course on its own did not improve MH symptoms relative to control at 18 months (Andrews et al., 2022). Recent meta-analyses similarly suggest negligible or small effects of universal school-based CBT prevention programs for emotional problems (Caldwell et al., 2019; Shelemy, Harvey, & Waite, 2020; Werner-Seidler et al., 2021). No studies to date have examined Climate Schools' effects on *co-occurring* MH and SU concerns.

PreVenture is a manualized, brief, group-based personality-targeted (tier 2) SU prevention program focused on specific inhibited traits (hopelessness and anxiety sensitivity) and disinhibited traits (impulsivity and sensation seeking) (Edalati & Conrod, 2019). These traits have been shown to predict different age of onset, motivations for SU, and comorbidity patterns (Conrod & Nikolaou, 2016). High-risk students are identified using the Substance Use Risk Profile Scale (SURPS; Woicik, Stewart, Pihl, & Conrod, 2009), whereby students scoring one standard deviation above their school's mean on particular subscales are invited to participate (~40% of school). The program includes two 90-min group-based sessions 1 week apart (four different groups for each personality trait), often conducted during school hours and facilitated by psychologists or trained school personnel. The groups are tailored to specific personality risk factors and are grounded in psychoeducation, motivational enhancement therapy, and CBT.

PreVenture has been evaluated in five trials across the UK, Netherlands, Australia, and Canada involving over 4000 students and 80 schools demonstrating positive alcohol-related outcomes (e.g. delayed onset of initiation, less coping motives, reduced quantity and frequency of drinking and alcohol-related problems) and improvements in emotional and behavioral symptoms (e.g. suicidal ideation, depression, anxiety, panic, conduct, victimization, and bullying) (Debenham et al., 2021; Edalati & Conrod, 2019; Grummitt et al., 2022; Lynch et al., 2023; Newton et al., 2020, 2022c; Slade et al., 2021). Most studies show protective effects up to 3 years (Debenham et al., 2021; Edalati & Conrod, 2019; Grummitt et al., 2022; Lynch et al., 2023; Newton et al., 2020; Slade et al., 2021), though a recent study showed benefits for alcohol-related outcomes up to 7 years (Newton et al., 2022b, 2022c). Perrier-Ménard, Castellanos-Ryan, O'Leary-Barrett, Girard, and Conrod (2017) found that higher emotional symptoms at baseline did not impact treatment outcomes, but higher behavioral symptoms at baseline did result in greater alcohol-related short-term benefits. Lynch et al. (2023) also found PreVenture to reduce growth in general psychopathology (SU and MH symptoms) over 3 years. While no studies have yet explored differential program effects on single *v.* dual alcohol use and psychological distress, these studies suggest differential impacts of this program on adolescent comorbidity.

By leveraging two, large, world-first longitudinal prevention trials in Australia, the primary objective of this study was to determine the presence and magnitude of 3-year prevention effects on the development of co-occurring alcohol use and psychological distress (a construct capturing depressive and anxiety symptoms). Second, this study explores prevention effects on alcohol use *without* psychological distress and psychological distress *without* co-use of alcohol. We hypothesized that students receiving both Climate SU and MH courses combined (CSC), PreVenture alone, and both Climate SU and PreVenture combined (CAP) would have a lower risk of experiencing co-occurring problems given these programs focus on skills and content related to both SU and MH problems. The tertiary objective was to determine whether sex moderates these associations given males are more likely to experience SU problems, females are more likely to experience MH problems with and without co-occurring SU (Erol & Karpyak, 2015; Lawrence et al., 2015), and several previous analyses of these programs found differences in prevention mechanisms, targets, or effects for females *v.* males (Debenham et al., 2021; Vogl et al., 2009).

## Methods

### Study design and sample

The study sample was derived from harmonizing data across the CSC and CAP Australian four-arm cluster randomized trials. Both trials had primary end points at 30–36 months post-baseline. Study protocols are published elsewhere (Newton et al., 2012; Teesson et al., 2014). Both trials were designed to primarily examine the effectiveness of various programs on reducing the initiation and escalation of alcohol use and, either primarily or secondarily, reducing rates and symptoms of anxiety and depression. *A priori* analyses focused on SU and MH outcomes as distinct outcomes. Thus, the current approach to analyze prevention effects on *co-occurring* alcohol and psychological distress is directly related, though distinct, from original study objectives.

**Figure 1.**
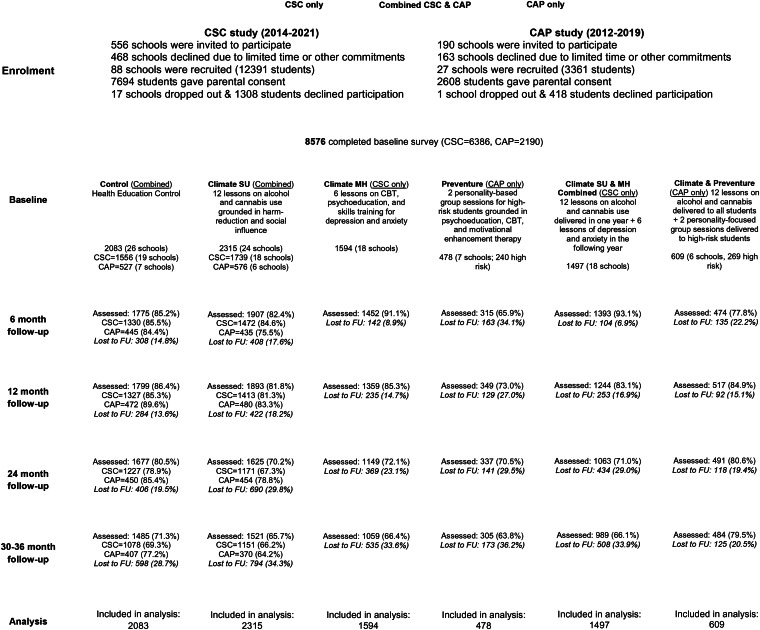
CONSORT participant flow.

Cluster randomization at the school level was used via statistical or online randomization software. All students within participating schools who provided written parental and student informed consent were included. The CAP trial included 2190 students in 26 schools while the CSC trial included 6386 students in 71 schools. Combined data include the whole sample of 8576 students in 97 schools receiving either health education as usual or a prevention program (see Fig. 1 for participant flow). Five prevention programs are compared: (1) Climate SU; (2) Climate MH; (3) PreVenture; (4) Climate Schools Combined (all students received both Climate SU and Climate MH); and (5) Climate SU and PreVenture (all students received Climate SU and high-risk students additionally received PreVenture). Students were assessed at baseline and 6, 12, 24, and 30 or 36 months (from ages ~13 to 16). Using an intention-to-treat approach, all students across participating schools are included in this analysis, even if they did not receive the intervention – for example, all students in schools that received PreVenture are included, not just the high-risk subgroup receiving PreVenture.

### Measures

#### Time

Assessment waves were coded as continuous months since baseline, including 0, 6, 12, 24, and 30 or 36 months.

#### Psychological distress

Both studies measured psychological distress using the Kessler-6 (K6), a 6-item measure on the frequency of feeling nervous, hopeless, restless or fidgety, depressed, that everything was an effort, and worthless over the past 4 weeks (Kessler et al., 2002). Response options were rated from 0 ‘*none of the time*’ to 4 ‘*all of the time*’. All responses were summed, resulting in a single total score ranging from 0 to 24, with higher scores indicating greater distress. In the current sample, the K6 demonstrated high internal consistency (*α* = 0.85–0.93) and invariance across time within the full harmonized sample, as well as stratified by study and sex (see online Supplementary materials for details). A score of ⩾8 was used to indicate moderate-to-serious distress (Boak, Elton-Marshall, Mann, & Hamilton, 2020).

#### Past 6 month alcohol use

In both studies, students were asked, ‘*How often did you have a standard alcoholic drink in the past 6 months?*’ with response options of never, less than monthly, once a month, 2–3 times a month, weekly, or daily or almost daily. In 2013, the average age of alcohol initiation among young Australians was 15.6 years of age and has since risen (Australian Institute of Health and Welfare, 2020). Therefore, any past 6-month alcohol use ⩽30–36 months (average ages across arms 14.7–15.3) is considered age-related risky alcohol consumption. As such, a binary variable of any past 6-month alcohol use was used (0 = no, 1 = yes).

#### Co-occurring alcohol use and distress quadrants

**Figure 2.**
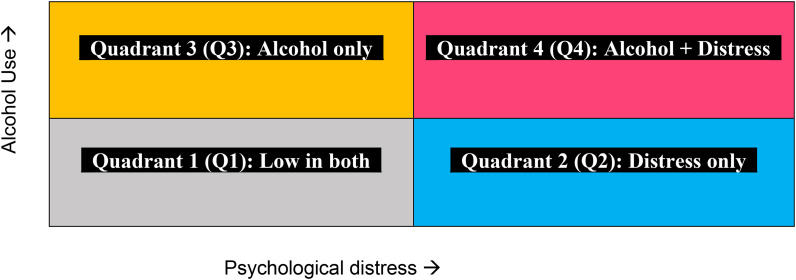
Visual depiction of the four-quadrants of co-occurrence.

The primary outcome in this study is a four-category multinomial variable based on the adapted 4-quadrant model of co-occurring problems created by combining the presence/absence of moderate-to-serious psychological distress and any alcohol use (Halladay et al., 2020). This variable includes youth reporting: Distress only (Q2: K6 ⩾ 8 and no past 6 month alcohol use), Alcohol only (Q3: alcohol use and K6 < 8), None (Q1: reference) (see Fig. 2). Generally average total distress scores were similar for Q1 (mean = 2.9 [range across time 2.3–3.4] and Q3 (mean = 2.8 [2.3–3.4]) and slightly higher in Q4 (mean = 14.3 [13.0–15.2]) versus Q2 (mean = 12.6 [11.8–13.7]). The frequency of alcohol use was higher for those in Q4 (mean = 3.2 [2.4–3.6]) Versus Q2 (mean = 1.7 [1.5–2.2]) where frequency of alcohol use was coded as 0 = never, 0.5 = less than monthly, 1 = once a month, 2.5 = 2–3 times a month, 4 = weekly, 30 = daily or almost daily. To note, while the categorical 4-quadrant classification was multiply imputed, the total distress scores and frequency of alcohol use were not. Thus, these averages are based on complete data only.

#### Demographics and covariates

School-level demographics based on the sampling design include study (CSC, CAP), state (New South Wales, Queensland, Western Australia), and school type (Public, Private, Catholic). Student-level covariates include baseline age, country of birth (previously related to student SU and MH; Halladay et al., 2022), sex (‘*Are you male or female*’), and baseline student personality traits measured by SURPS subscales (Woicik et al., 2009). Further, behavioral symptoms were measured through the hyperactivity and conduct problems subscales from the Strengths and Difficulties Questionnaire (SDQ) (Goodman, 2001). While the SDQ was not asked across CAP public schools (*k* = 9, *n* = 554 [25.3%]), controlling for externalizing symptoms is important when examining relationships between SU and MH (Hussong, Ennett, Cox, & Haroon, 2017), and thus scores were imputed and incorporated into fully adjusted models that should be interpreted as a sensitivity analysis.

### Analysis plan

As per published protocols, multilevel mixed-effects regression models were used following intention-to-treat procedures, whereby all participants were analyzed in the groups to which they were originally allocated during randomization. Multilevel multinomial generalized linear mixed models were used to predict membership in the co-occurring quadrants across time (reference = Q1). Models were also run with Q4 as the reference category and with time treated categorically (see online Supplementary materials). Specifically, two-level multinomial regressions with standard error adjustments accounting for school clustering were conducted in Stata 18 using *xtmlogit*. First, missing data were explored. In multivariable models, there were no significant differences in retention across programs or baseline distress or co-occurrence. However, those reporting alcohol use only at baseline, being male, being born outside of Australia, and higher baseline conduct symptoms, sensation seeking, negative thinking were more likely to have missing follow-up data (see online Supplementary materials). Missing data were addressed, using these predictors of missingness, through multilevel multiple imputation using BLIMP's fully Bayesian model-based approach with a full condition Metropolis Sampler (see online Supplementary materials for details) (Keller & Enders, 2021). Models were estimated separately for each imputed dataset (20 imputations) with effects pooled across imputations using Rubin's rules (*mi estimate*, *cmdok*). To note, main models using complete data only or inverse probability weighting to account for missingness were also estimated, with results available in the online Supplementary materials. A series of models were estimated including: model 1, regressing quadrant assignment on primary covariates (study and sex), program, and time; model 2, adding time × program interaction (i.e. differences by program over time); model 3, adding sex × time × program interaction (i.e. exploration of sex-differences); and model 4, further adjusting for other covariates related to baseline differences. Non-independence of repeated measures within students and schools was addressed through student-level random intercepts and standard error adjustments accounting for school clustering. The program main effects represent program differences in the risk of being in each quadrant at baseline. The linear time main effect represents relative average 1-month change in the risk of the outcome(s) for health education control. (Note: the inclusion of quadratic time effects worsened model fit and were not significant, and thus not retained; see online Supplementary materials.) The program × linear time interactions reflect program differences in the relative average 1-month change in risk of the outcome, adjusted for baseline. The program × time effects and respective confidence intervals were multiplied by 12, 24, and 36 months to provide estimated prevention effects at yearly increments. To guide interpretation, relative risk ratios (RRs) of 0.98/1.02, 0.71/1.38, 0.43/2.16, and 0.25/3.22 are considered very small, small, medium, and large, respectively (for outcomes ~10% prevalence); notably, even very small effects are meaningful at a population level (Matthay et al., 2021).

## Results

**Figure 3.**
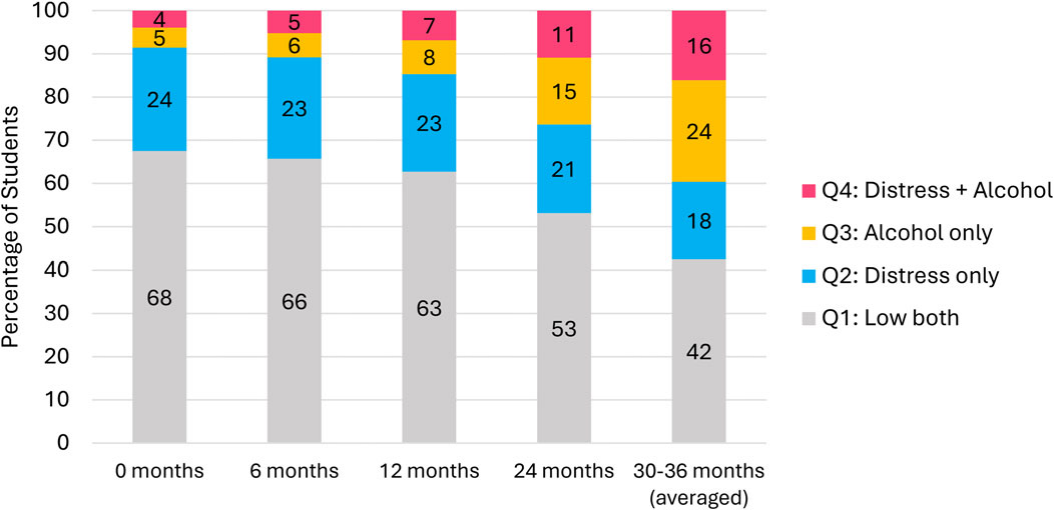
Unadjusted pooled prevalence of four-quadrants over time. *Note*: Distress = K6 ⩾ 8, alcohol = any past 6-month alcohol use.

Overall, 51.7% of the harmonized sample was female, 82.8% born in Australia, 45.6% were from government/public schools, 25.8% from independent/private schools, and 28.6% from Catholic schools. Almost half (44.2%) of the sample were from NSW, 30.5% from QLD, and 25.3% from WA (see Table 1). The unadjusted prevalence of *co-occurring* distress and alcohol (Q4) increased from 4% to 16%, alcohol only (Q3) increased from 5% to 24%, and distress only (Q2) decreased from 24% to 18% between baseline and 30–36 months (see Fig. 3). In the fully adjusted model (see Table 2), the prevalence of adolescents reporting elevations in either distress and/or alcohol use (Q2, Q3, Q4) in the control group increased overtime, though more-so for alcohol-related outcomes (Q3, Q4). Additionally, females had a higher risk of being in distress-related outcome groups (Q2, Q4) compared to males.

**Table 1.** Descriptive statistics

	Control (*n* = 2083; 24.3%)	Climate SU (*n* = 2315; 27.0%)	Climate MH (*n* = 1594; 18.6%)	PreVenture (*n* = 478; 5.6%)	CSC (*n* = 1497; 17.5%)	CAP (*n* = 609; 7.1%)
Age at baseline	13.5 (0.4)	13.5 (0.5)	13.6 (0.5)	13.5 (0.4)	13.5 (0.6)	13.4 (0.4)
Male	33.2%	50.4%	39.3%	82.0%	52.1%	79.3%
Female	66.8%	49.6%	60.7%	18.0%	47.9%	20.7%
Australia born	84.2%	83.1%	82.3%	82.6%	80.1%	85.7%
Other English-speaking country	5.8%	5.7%	7.8%	10.9%	7.6%	8.5%
Non-English-speaking country	10.1%	11.2%	9.9%	6.5%	12.3%	5.8%
Hyperactivity symptoms	3.2 (2.4)	3.4 (2.6)	3.3 (2.4)	3.7 (2.8)	3.4 (2.2)	3.3 (2.5)
Conduct symptoms	1.7 (2)	2.1 (2.6)	1.9 (2)	2.6 (2.6)	2.1 (2.1)	2.1 (2.2)
Anxiety sensitivity	11.2 (3.2)	11.2 (3.2)	10.9 (3.4)	11.4 (2.8)	10.7 (3.3)	11.6 (2.6)
Impulsivity	10.7 (3.1)	11.1 (3.2)	10.9 (3.4)	12.2 (2.7)	11 (3.3)	11.6 (2.8)
Sensation seeking	15.9 (3.6)	15.8 (3.7)	16 (4.1)	16.6 (3.1)	15.8 (3.9)	16.3 (3.3)
Negative thinking	12.4 (4.2)	12.7 (4.1)	12.6 (4.3)	12.2 (3.2)	13 (4.4)	11.5 (3.2)

**Table 2.** Multilevel multinomial logistic regression models pooled across 20 imputations

Reference = No alcohol + low/no distress	Basic model RR (95% confidence interval); *p* value			Adjusted model RR (95% confidence interval); *p* value		
	Distress only	Alcohol only	Distress + alcohol	Distress only	Alcohol only	Distress + alcohol
Female sex (ref = male)	2.11 (1.79–2.48); 0	0.74 (0.5–1.09); 0.13	1.98 (1.47–2.69); 0	1.92 (1.70–2.17); 0	0.97 (0.65–1.44); 0.885	2.12 (1.60–2.81); 0
CAP study (ref = CSC)	1.21 (0.91–1.61); 0.192	2.9 (1.47–5.72); 0.002	4.03 (2.45–6.62); 0	1.06 (0.88–1.28); 0.53	1.13 (1.11–1.15); 0	3.19 (1.98–5.12); 0
Time (1 month)	1.01 (1–1.01); 0.143	1.13 (1.11–1.15); 0	1.11 (1.09–1.13); 0	1.01 (1–1.02); 0.021	1.13 (1.11–1.15); 0	1.11 (1.09–1.13); 0
Climate SU (ref = control)	1.21 (0.9–1.62); 0.209	2.03 (0.9–4.61); 0.09	2.37 (1.16–4.82); 0.017	1.02 (0.83–1.27); 0.837	1.88 (0.86–4.13); 0.115	1.83 (1.04–3.24); 0.037
Climate MH (ref = control)	1.14 (0.81–1.61); 0.449	2.06 (0.82–5.22); 0.126	2.81 (1.23–6.39); 0.014	1.03 (0.81–1.29); 0.827	1.8 (0.74–4.35); 0.195	2.16 (1.08–4.34); 0.03
PreVenture (ref = control)	1.17 (0.79–1.73); 0.423	3.1 (1.17–8.17); 0.022	3.63 (1.61–8.16); 0.002	1.07 (0.79–1.45); 0.639	2.35 (0.92–6.01); 0.073	2.53 (1.2–5.35); 0.015
CSC (ref = control)	1.12 (0.78–1.63); 0.534	3.19 (1.26–8.09); 0.015	3.49 (1.3–9.35); 0.013	0.92 (0.73–1.14); 0.431	2.66 (1.12–6.29); 0.026	2.27 (0.99–5.25); 0.054
CAP (ref = control)	0.75 (0.44–1.29); 0.296	2.54 (1.03–6.25); 0.042	1.39 (0.65–2.97); 0.395	0.88 (0.6–1.3); 0.53	2.47 (1.05–5.82); 0.039	1.49 (0.78–2.83); 0.228
Climate SU × month	1 (0.99–1.02); 0.337	**0.98 (0.95–0.997); 0.024**	0.99 (0.97–1.01); 0.238	1 (0.99–1.02); 0.357	**0.98 (0.96–0.997); 0.026**	0.99 (0.97–1.01); 0.203
Climate MH × month	1 (0.99–1.01); 0.907	1 (0.97–1.02); 0.771	0.99 (0.97–1.01); 0.296	1 (0.99–1.01); 0.946	1 (0.97–1.02); 0.817	0.99 (0.97–1.01); 0.3
PreVenture × month	0.99 (0.97–1); 0.102	**0.96 (0.93–0.98); 0.002**	**0.96 (0.93–0.98); 0.001**	0.99 (0.97–1); 0.104	**0.96 (0.93–0.99); 0.003**	**0.96 (0.93–0.98); 0.002**
CSC × month	0.99 (0.98–1); 0.192	**0.96 (0.94–0.98); 0**	**0.97 (0.95–1); 0.021**	0.99 (0.98–1); 0.168	**0.96 (0.94–0.98); 0**	**0.97 (0.95–1); 0.017**
CAP × month	1.01 (0.99–1.02); 0.384	**0.97 (0.94–0.99); 0.009**	0.98 (0.95–1.01); 0.131	1.01 (0.99–1.02); 0.419	**0.97 (0.94–0.99); 0.015**	0.98 (0.96–1.01); 0.168
Other English-speaking country				1.13 (0.94–1.37); 0.199	1.15 (0.83–1.57); 0.4	1.57 (1.18–2.1); 0.002
Other non-English-speaking Country				1.3 (1.1–1.54); 0.002	0.32 (0.22–0.47); 0	0.36 (0.24–0.54); 0
SDQ hyperactivity				1.23 (1.19–1.26); 0	1.05 (1–1.1); 0.052	1.38 (1.31–1.45); 0
SDQ conduct				1.08 (1.04–1.12); 0	1.09 (1.04–1.15); 0.001	1.21 (1.14–1.27); 0
Anxiety sensitivity				1.12 (1.1–1.15); 0	0.93 (0.91–0.96); 0	1.04 (1.01–1.07); 0.013
Impulsivity				1.03 (1–1.06); 0.033	1.09 (1.05–1.12); 0	1.02 (0.98–1.07); 0.302
Sensation seeking				1.02 (1–1.03); 0.083	1.18 (1.15–1.22); 0	1.19 (1.15–1.22); 0
Negative thinking				1.21 (1.19–1.23); 0	1.13 (1.10–1.16); 0	1.29 (1.26–1.33); 0

*Note*: Bold indicates program × time effects with a *p* value <0.05.

There were significant program × time interactions that were retained after adjusting for baseline personality and behavioral variables (Tables 2 and 3). After adjusting for baseline differences, Climate SU, PreVenture, CAP, and CSC all yielded significant protective effects on at least one alcohol and/or distress related outcome category. Related to co-occurrence (Q4), PreVenture, and CSC reduced the risk of adolescents reporting co-occurring alcohol use and distress by 36 months post-baseline by 78% (large effect) and 63% (medium) respectively. For alcohol only (Q3), all programs other than Climate MH reduced the risk of adolescents reporting alcohol use without co-occurring distress over time. Specifically, Climate SU had a relative risk reduction of 58% (medium), PreVenture 77% (large), CSC 79% (large), and CAP 69% (medium-large) by 36 months post-baseline. For distress only (Q2), no prevention program had a significant effect over time. When comparisons were made with adolescents experiencing co-occurrence (Q4), the program × time interactions were not significantly different for those reporting alcohol only (Q3) though Climate SU, PreVenture, and CAP yielded a significantly higher prevalence of adolescents experiencing distress only (Q2), while only PreVenture and CSC increased the prevalence of those indicating low levels of both alcohol and distress (Q1) compared to co-occurrence (see online Supplementary materials). Similar program effects were found when using complete data only, inverse probability weighting, or when time was treated categorically (see online Supplementary materials). There was also no evidence of significant differences by sex (see online Supplementary materials for details).

**Table 3.** Estimated relative risk at 12, 24, and 36 months (fully adjusted model)

	Distress only			Alcohol only			Distress + alcohol		
	12 months	24 months	36 months	12 months	24 months	36 months	12 months	24 months	36 months
Climate SU	1.06 (0.94–1.2)	1.13 (0.88–1.45)	1.19 (0.82–1.74)	**0.75 (0.58–0.97)**	**0.56 (0.34–0.93)**	**0.42 (0.19–0.9)**	0.87 (0.7–1.08)	0.75 (0.48–1.17)	0.65 (0.34–1.26)
Climate MH	1 (0.88–1.13)	0.99 (0.77–1.27)	0.99 (0.68–1.44)	0.97 (0.73–1.29)	0.93 (0.53–1.66)	0.9 (0.38–2.13)	0.87 (0.67–1.13)	0.76 (0.45–1.28)	0.66 (0.3–1.45)
PreVenture	0.86 (0.71–1.03)	0.74 (0.51–1.07)	0.63 (0.36–1.1)	**0.61 (0.44–0.85)**	**0.37 (0.19–0.71)**	**0.23 (0.09–0.6)**	**0.6 (0.44–0.83)**	**0.37 (0.2–0.68)**	**0.22 (0.09–0.56)**
CSC	0.91 (0.8–1.04)	0.83 (0.64–1.08)	0.76 (0.51–1.12)	**0.6 (0.46–0.78)**	**0.36 (0.21–0.61)**	**0.21 (0.1–0.47)**	**0.72 (0.55–0.94)**	**0.52 (0.3–0.89)**	**0.37 (0.17–0.84)**
CAP	1.08 (0.89–1.31)	1.17 (0.8–1.71)	1.27 (0.72–2.24)	**0.68 (0.49–0.93)**	**0.46 (0.25–0.86)**	**0.31 (0.12–0.8)**	0.8 (0.58–1.1)	0.64 (0.33–1.21)	0.51 (0.19–1.33)

*Note*: Bold indicates program × time effects with a *p* value <0.05.

## Conclusions

This study evaluated ~3-year outcomes of five school-based prevention programs evaluated in two world-first Australian cluster-based randomized controlled trials with a combined sample of over 8500 adolescents. Two of these programs reduced the risk of adolescents experiencing *co-occurring* early alcohol use and psychological distress (Q4), with the CSC program yielding moderate-sized effects and PreVenture yielding large effects. Of the four programs with an intended goal of preventing SU, all reduced the risk of adolescents reporting early alcohol use without co-occurring psychological distress (Q3) across the ~3-year observation period. None of the programs had a significant impact on the risk of students reporting distress without co-occurring alcohol use (Q2) over time. There were no meaningful sex differences in program effects over time, though females were two times more likely to report distress with (Q4) and without (Q2) co-occurring alcohol use. Overall, two available school-based programs reduce the risk of early alcohol use with *and* without co-occurring psychological distress, four reduce the risk of early adolescent alcohol use on its own, and none currently appear effective for preventing distress alone. Given no programs helped reduce the risk of psychological distress only and only two reduced the risk of co-occurring problems (outcomes twice as likely among females), existing programs may indirectly be less likely to benefit female adolescents.

Students in schools implementing the PreVenture program alone saw large reductions in the risk of co-occurring early alcohol use and distress over time (Q4). While this study explored effects for all students – even those not directly participating in the selective PreVenture program – these findings align with prior analyses focused on high-risk students only. Lynch et al. (2023) found a slower growth in general psychopathology over 36 months for high-risk students, defined as a higher-order construct representing the correlation among fear, distress, alcohol harms, and conduct and inattention symptoms. Further, Perrier-Ménard et al. (2017) found PreVenture effective in preventing alcohol-related outcomes over 24 months in high-risk students, irrespective of pre-existing emotional symptoms. These findings collectively indicate PreVenture's efficacy in preventing co-occurring problems. However, combining PreVenture with Climate SU (CAP) did not prevent co-occurrence in the current study. Slade et al. (2021) similarly found combining programs did not yield additional benefits in alcohol prevention above the stand-alone programs; by 3 years, Slade et al. (2021) reported that compared to control, the RR reduction in odds of drinking among adolescents in the Climate SU program was 74%, PreVenture 83%, and CAP 70%. PreVenture was designed to target SU risk profiles and teach skills relevant to both SU and MH problems, and current evidence suggests that PreVenture on its own is effective at preventing these co-occurring SU and MH problems.

The combined Climate SU and MH programs (CSC) were the second condition to prevent co-occurrence, though these programs did not prevent co-occurrence when implemented separately. The main outcomes paper for the CSC trial (Teesson et al., 2020) similarly found that compared to control, adolescents participating in the combined program had a slower growth in drinking and lower odds of drinking by 30 months than those participating in control (~75% relative reduction in odds), Climate SU alone (~56% reduction), or Climate MH alone (~80% reduction). At 30 months, it was also found that symptoms of depression and anxiety were lower in the combined group compared to Climate MH and, while non-significant, lower point estimates when compared to Climate SU and control (Teesson et al., 2020). As such, both the current study and the paper reporting on the main outcomes support the finding that the combined program had greater prevention effects related to alcohol use, MH, and their co-occurrence than stand-alone programs. By combining these programs (CSC) adolescents may be equipped with the insight and skills to reduce the use of alcohol for coping purposes and/or target other drivers of alcohol use and distress that are unique to adolescents at risk for experiencing co-occurring problems.

The risk of adolescents reporting early alcohol use only (Q3) was reduced among those participating in Climate SU, CSC, PreVenture, or CAP. This aligns with prior studies showing prevention effects on various alcohol-related outcomes among adolescents participating in Climate SU (Champion et al., 2016; Newton et al., 2009a, 2009b, 2010, 2022a, 2022c; Slade et al., 2021; Teesson et al., 2017, 2020; Vogl et al., 2009), PreVenture (Edalati & Conrod, 2019; Newton et al., 2022a, 2022b; Slade et al., 2021), CSC (Teesson et al., 2020), or CAP (Slade et al., 2021). As such, universal tier 1 curricular approaches focused on harm-reduction and social-influence (Newton et al., 2011) and targeted tier 2 approaches for high-risk students using personality tailored psychoeducation, motivational enhancement therapy, and CBT (Edalati & Conrod, 2019) appear to provide the knowledge and skills for adolescents without distress to avoid or delay alcohol use initiation. Notably, both CSC and PreVenture reduced the risk of students reporting alcohol use without (Q3) and with (Q4) co-occurring psychological distress when compared to no/low alcohol and distress (Q1), suggesting these programs have generalized protective effects for early alcohol use while others may yield benefits specific to lower risk adolescents. However, there were no significant differences in program effects on alcohol only (Q3) when directly compared to co-occurrence (Q4), and thus programs may be similarly effective for preventing alcohol use with or without co-occurring distress.

No program significantly impacted adolescents reporting psychological distress without co-occurring alcohol use (Q2), including the Climate MH course that was designed to prevent emotional problems. The main CSC outcomes paper similarly found negligible benefits of Climate MH when implemented alone (Teesson et al., 2020) and Andrews et al. (2022) found a small, transient, iatrogenic effect on internalizing symptoms, though this was found using a different measure of emotional problems. This finding is in line with evidence suggesting universal school-based CBT programs do not meaningfully and consistently reduce emotional problems (Caldwell et al., 2019; Shelemy et al., 2020; Werner-Seidler et al., 2021). While reasons for this lack of benefit are poorly understood, one hypothesis is that these programs may unintentionally encourage rumination through changes in self context or peer influence (Foulkes & Stringaris, 2023). It is also possible that current programs are not intervening early enough to prevent distress, or may not be observing cohorts long enough to see preventive effects for depression, which peaks in young adulthood (Solmi et al., 2021). However, other evaluated programs that were not designed to prevent emotional problems on their own, have previously suggested possible secondary prevention. For example, an earlier trial found the Climate SU reduced psychological distress at 12-month follow-up compared to control, though most students had scores below 8 (the cut-off for moderate distress) (Newton, Andrews, Champion, & Teesson, 2014). Further, while PreVenture has shown benefits on various emotional problems (i.e. suicidality, distress, anxiety, and depressive symptoms) (Edalati & Conrod, 2019; Grummitt et al., 2022; Lynch et al., 2023; Newton et al., 2020), it is possible these effects may be explained by non-specific broader reductions in general psychopathology (i.e. protective effects for distress may be restricted to those experiencing some indication of comorbidity) (Lynch et al., 2023) or may only emerge when restricting analyses to the high-risk youth directly participating in the intervention (i.e. effects previously noted may have been dampened by analyzing all students regardless of PreVenture participation) (Newton et al., 2020). There was some indication in the current results that reduced likelihoods of adolescents experiencing co-occurrence (Q4) may be explained by more adolescents reporting psychological distress without alcohol use (Q2) when students participated in Climate SU, PreVenture, and CAP. Results related to PreVenture and CSC further suggested that lower co-occurrence (Q4) may be additionally explained by more adolescents reporting low in both (Q1). Overall, none of the evaluated programs in this study appear to target mechanisms that universally prevent psychological distress in the whole student population that occurs among adolescents who do not initiate alcohol use early.

While the sample was not meant to be representative, the combined sample is a large and diverse sample of adolescents in Australia. As often seen in school-based research, school participation was relatively low among initial schools invited to the studies and thus results should be generalized with caution. External validity is strengthened by the diversity of schools in the sample, including private, government, and Catholic secondary schools across Australia. The cluster randomized controlled trial design limits risk of confounding, which was further mitigated in fully adjusted multilevel models adjusting for clustering and any notable baseline differences between intervention arms. There remains risk of residual confounding, particularly due to use of other substances (not included due to inconsistent measurement across studies and schools) and behavioral symptoms (imputed across CAP public schools). There were chance imbalances in the allocation of males and females across study arms, particularly in the CAP trial, though all models were adjusted for sex and sex did not appear to moderate effects. Any unmeasured confounding variables could also be unbalanced at baseline. Gender was also inconsistently measured, so we were unable to tease apart differences due to biological sex from sociocultural gender. Further, while retention was good with 85% of adolescents completing more than 60% of all key study variables across time points, missing data is still a concern. To mitigate the risk of bias due to missing data, comprehensive contemporary missing data analysis strategies were used. Notably, main findings based on pooled imputed data are similar (though more conservative) to complete case findings and models using inverse probability weighting (see online Supplementary materials). Lastly, non-blinding of schools may have resulted in differential implementation of alternative school-wide prevention initiatives over the observation period; however, this is more likely to occur in control schools (since they were not implementing programs) and thus may have resulted in underestimation of effects. Notably, school clustering was accounted for in all models which will partially adjust for differential interventions or other characteristics across schools. Lastly, our study relied on self-reported SU, which is a potential limitation, though data were obtained from structured and validated instruments commonly used and well-accepted in SU prevention research (Del Boca & Darkes, 2003; Smith, McCarthy, & Goldman, 1995). Notably, the assessment protocols employed all components known to maximize reliable self-report by adolescents (Brener, Billy, & Grady, 2003).

This study demonstrated that the selective prevention program PreVenture, and the combined universal prevention program Climate Schools: Alcohol and Cannabis delivered with Climate Schools: Mental Health, reduced the risk of adolescents engaging in early alcohol use with and without co-occurring elevations in psychological distress. The other evaluated programs only appeared effective for alcohol use that occurs without co-occurring distress, and thus these programs may be inadvertently leaving behind adolescents with or at risk for MH problems. In sum: (1) evidence-based programs exist to reduce the risk of early alcohol use; (2) some, but not all, address co-occurring alcohol use and distress; and (3) new and/or adapted programs are needed to address distress that occurs without co-occurring alcohol use. This suggests that psychological distress occurring in the context of early adolescent alcohol use may be a different type of distress than that which occurs without early alcohol use. Alcohol use in the context of distress may also be a different type of use than that which occurs without distress. These different ‘types’ of alcohol use and distress appear to respond differently to prevention programs, and thus may require different prevention strategies; however, more research is needed to deepen our understanding of the developmental sequencing of comorbidity, universal and specific risk and protective factors related to these different patterns of problems, and ‘type’ specific prevention considerations across adolescent development. Additionally, future practice and research should focus on scaling up effective programs to reduce the significant burden of adolescent SU with and without co-occurring MH problems while exploring new, personalized prevention approaches targeting different profiles of co-occurrence.

## Supporting information

Halladay et al. supplementary material Halladay et al. supplementary material

## Supplementary material

The supplementary material for this article can be found at https://doi.org/10.1017/S0033291724002897.
