# Supplementary material for: Do school-based prevention programs impact co-occurring alcohol use and psychological distress during adolescence?
Source: Psychol Med. 2024 Dec 23;54(16):4725–35. doi: 10.1017/S0033291724002897 (PMC11779555; doi:10.1017/S0033291724002897)
Supplement: Halladay et al. supplementary material [file S0033291724002897sup001.docx]

# Do school-based prevention programs impact co-occurring alcohol use and psychological distress during adolescence?

**SUPPLEMENTARY MATERIALS**

**Supplementary Materials 1 Table 1**. K6 measurement invariance

**Supplementary Materials 2**. Missing data

- Supplementary Materials 2, Table 2.1. Regressions predicting continued engagement

**Supplementary Materials 3.** Selecting model covariates and baseline differences

- Supplementary Materials 3, Table 3.1. Final single-level multivariable multinomial logistic regression predicting baseline differences between prevention groups

**Supplementary Table 4.** Comparing models with time as a linear and quadratic effect

**Supplementary Table 5**. Main model with Q4 as the reference group

**Supplementary Table 6**. Sensitivity analyses for missing data

**Supplementary Table 7**. Main analysis with categorical time

**Supplementary Table 8.** Differential program effects by sex^1^

**Supplementary Materials Figure 2.** Model-predicted probabilities (Imputation #20, Model 1)

**Supplementary Table 9**. Predicted probabilities of quadrants by group across time

**Supplementary Table 1.** K6 invariance using robust maximum likelihood estimation (0, 6, 12, 24, and 30-36 months)^1^

|  | CFI | RMSEA (90% CI) | SRMR | ΔCFI | ΔRMSEA | ΔSRMR |
| --- | --- | --- | --- | --- | --- | --- |
| Full (*n*=8,543) | | | | | | |
| Configural | 0.958 | 0.040 (0.039-0.0341) | 0.031 |  |  |  |
| Metric | 0.954 | 0.0341 (0.040-0.042) | 0.034 | -0.004 | -0.0059 | 0.003 |
| Scalar | 0.951 | 0.041 (0.040-0.042) | 0.036 | -0.003 | 0.0069 | 0.002 |
| CSC (*n*=6,365) | | | | | | |
| Configural | 0.960 | 0.039 (0.038-0.041) | 0.031 |  |  |  |
| Metric | 0.955 | 0.040 (0.039-0.042) | 0.035 | -0.005 | 0.001 | 0.004 |
| Scalar | 0.949 | 0.042 (0.041-0.043) | 0.038 | -0.006 | 0.002 | 0.003 |
| CAP (*n*=2,178) | | | | | | |
| Configural | 0.952 | 0.043 (0.041-0.045) | 0.036 |  |  |  |
| Metric | 0.950 | 0.042 (0.040-0.044) | 0.038 | -0.002 | -0.001 | 0.002 |
| Scalar | 0.946 | 0.043 (0.041-0.045) | 0.039 | -0.004 | 0.001 | 0.001 |
| Female (*n*=4,425) | | | | | | |
| Configural | 0.960 | 0.041 (0.039-0.042) | 0.035 |  |  |  |
| Metric | 0.957 | 0.041 (0.040-0.043) | 0.040 | -0.003 | 0 | 0.005 |
| Scalar | 0.955 | 0.041 (0.040-0.042) | 0.040 | -0.002 | 0 | 0 |
| Male (*n=*4,116) | | | | | | |
| Configural | 0.955 | 0.039 (0.038-0.041) | 0.030 |  |  |  |
| Metric | 0.953 | 0.039 (0.038-0.041) | 0.032 | -0.003 | 0 | 0.005 |
| Scalar | 0.944 | 0.041 (0.040-0.043) | 0.036 | -0.002 | 0 | 0 |

Note: Tenability of measurement invariance overtime was established by meeting at least two out of overall model-fit thresholds including CFI>=0.95, RMSEA<0.08, SRMR<0.08 and change in model fit estimates for large sample sizes of CFI >=-0.02, RMSEA<=0.03 [metric] and <=0.01 [scalar], and SRMR <0.03 were used.^1,2^

^1^Analyses done in Mplus 7

**1.** Chen FF. Sensitivity of Goodness of Fit Indexes to Lack of Measurement Invariance. *Structural Equation Modeling: A Multidisciplinary Journal.* 2007/07/31 2007;14(3):464-504.

**2.** Rutkowski L, Svetina D. Assessing the hypothesis of measurement invariance in the context of large-scale international surveys. *Educational and psychological measurement.* 2014;74(1):31-57.

**Supplementary Materials 2.** Missing data

All sum scores were pro-rated (i.e., person-mean substitution) for up to <=40% missing data. During follow-up periods (0-36 months), 85% of students had complete alcohol and psychological distress data across >= 60% of surveys (43% had complete responses over the 3 years). Predictors of study retention were obtained through a series of multilevel (2-level, students within schools) univariable linear models predicting the number of surveys with complete alcohol and psychological distress data from baseline to 30-36 months (0 to 5). All aforementioned covariates were explored. Covariates with p<0.05 in univariable models were retained for a multivariable regression, whereby all covariates were retained (regardless of p-values) as long as the variance inflation factor (VIF) was < 3.5. Missing data was then multiply imputed via three-level multilevel imputation using BLIMP, clustering by student repeated assessments and school (20 imputations) using a fully Bayesian model-based approach with a full condition Metropolis Sampler.^56^ The imputation model included all variables in the final retention model and primary model effects (i.e., time × program effects, random intercepts).

**Supplementary Materials 2, Table 2.1.** Regressions predicting continued engagement^1^

| Baseline variables | **Continued engagement (Univariable)**  B (95% CI); p-value | **Continued engagement (multivariable)**  B (95% CI); p-value |
| --- | --- | --- |
| *Core analysis variables* |  |  |
| Baseline Quadrant |  |  |
| Psychological Distress Only | -0.2 (-0.25 to -0.14); <.0001 | -0.01 (-0.08 to 0.05); 0.7442 |
| Risky Alcohol Only | -0.87 (-1.24 to -0.5); <.0001 | -0.5 (-0.9 to -0.1); 0.0147 |
| Distress + Alcohol | -0.23 (-0.52 to 0.07); 0.1386 | -0.02 (-0.34 to 0.31); 0.9242 |
| CAP (vs. CSC) | -0.14 (-0.38 to 0.1); 0.2568 | *Not included* |
| Program |  |  |
| Climate SU | -0.3 (-0.59 to -0.01); 0.0399 | -0.25 (-0.52 to 0.03); 0.079 |
| Climate MH | -0.11 (-0.43 to 0.2); 0.4715 | -0.1 (-0.39 to 0.19); 0.5025 |
| PreVenture | -0.58 (-1.02 to -0.14); 0.0091 | -0.5 (-1 to 0); 0.0509 |
| CSC | -0.22 (-0.55 to 0.1); 0.1744 | -0.18 (-0.49 to 0.12); 0.232 |
| CAP | -0.37 (-0.83 to 0.09); 0.1126 | -0.04 (-0.53 to 0.46); 0.8836 |
| *Student and School Covariates* |  |  |
| Baseline age | -0.03 (-0.09 to 0.03); 0.3799 | *Not included* |
| Female sex (vs. male) | 0.17 (0.1 to 0.23); <.0001 | 0.15 (0.09 to 0.22); <.0001 |
| Born in other English-speaking country (vs. Australian Born) | -0.22 (-0.32 to -0.12); <.0001 | -0.2 (-0.3 to -0.1); <.0001 |
| Born in other non-English-speaking country (vs. Australian Born) | -0.14 (-0.22 to -0.06); 0.0009 | -0.11 (-0.19 to -0.02); 0.0138 |
| State - QLD (vs. NSW) | 0.08 (-0.18 to 0.33); 0.5453 | *Not included* |
| State - WA (vs. NSW) | -0.14 (-0.41 to 0.13); 0.3013 | *Not included* |
| School Type - Private (vs. Public) | 0 (-0.25 to 0.25); 0.9916 | *Not included* |
| School Type – Catholic (vs. Public) | 0.08 (-0.2 to 0.36); 0.5891 | *Not included* |
| *Auxiliary covariates* |  |  |
| SDQ Hyperactivity | -0.05 (-0.07 to -0.04); <.0001 | -0.01 (-0.03 to 0); 0.1112 |
| SDQ Conduct | -0.07 (-0.09 to -0.06); <.0001 | -0.04 (-0.05 to -0.02); <.0001 |
| SURPS Anxiety Sensitivity | -0.01 (-0.02 to 0); 0.0308 | 0 (-0.01 to 0.01); 0.6903 |
| SURPS Impulsivity | -0.04 (-0.05 to -0.03); <.0001 | -0.01 (-0.02 to 0.01); 0.3578 |
| SURPS Sensation Seeking | -0.01 (-0.02 to 0); 0.0015 | -0.01 (-0.02 to 0); 0.0053 |
| SURPS Negative Thinking | -0.03 (-0.04 to -0.03); <.0001 | -0.02 (-0.03 to -0.02); <.0001 |
| School ICC (empty model) | 16.9% |  |

^1^Analyses done using PROC GLIMMIX (RSPL) in SAS Enterprise Guide 7.1

**Supplementary Materials 3.** Selecting model covariates and baseline differences

**Adjusting for baseline differences**. To explore unintentional baseline differences between program arms, baseline covariates predicting program assignment were explored using similar modelling steps as was defined for retention. A series of multilevel univariable regression models were run. Covariates were retained if they had a p-value<0.05 and were not collinear in multivariable models (VIF<3.5). The final baseline differences model included sex, quadrant assignment, country of birth, hyperactivity symptoms, conduct symptoms, and SURPS subscale scores. Fully adjusted outcome models include these covariates predicting baseline differences.

**Supplementary Materials 3, Table 3.1.** Final single-level multivariable multinomial logistic regression predicting baseline differences between prevention treatment groups.^1^

| Ref=Control | Climate SU | Climate MH | PreVenture | CSC | CAP |
| --- | --- | --- | --- | --- | --- |
| Baseline Quadrant |  |  |  |  |  |
| Distress Only | 1.04 (0.87 to 1.24); 0.6966 | 1.02 (0.84 to 1.24); 0.8077 | 1.05 (0.74 to 1.49); 0.7651 | 0.85 (0.7 to 1.04); 0.1089 | 0.91 (0.68 to 1.22); 0.5318 |
| Risky Alcohol Only | 1.17 (0.8 to 1.71); 0.4215 | 1.16 (0.76 to 1.75); 0.4964 | 2.99 (1.85 to 4.83); <.0001 | 1.28 (0.86 to 1.9); 0.2251 | 2.98 (1.94 to 4.59); <.0001 |
| Distress + Alcohol | 1.42 (0.95 to 2.12); 0.0869 | 1.09 (0.69 to 1.7); 0.7194 | 3.12 (1.71 to 5.68); 0.0002 | 1.11 (0.72 to 1.72); 0.6316 | 2.74 (1.55 to 4.85); 0.0005 |
| Sex (female) | 0.44 (0.38 to 0.5); <.0001 | 0.81 (0.7 to 0.94); 0.0069 | 0.09 (0.06 to 0.12); <.0001 | 0.46 (0.4 to 0.54); <.0001 | 0.1 (0.08 to 0.13); <.0001 |
| Born in other English-speaking country (vs. Australian Born) | 0.96 (0.72 to 1.28); 0.7638 | 1.37 (1.03 to 1.83); 0.0299 | 1.47 (0.92 to 2.34); 0.1035 | 1.45 (1.09 to 1.93); 0.0117 | 1.44 (0.98 to 2.12); 0.0666 |
| Born in other non-English-speaking country | 1.15 (0.93 to 1.42); 0.1999 | 0.97 (0.77 to 1.24); 0.8268 | 0.41 (0.23 to 0.76); 0.0046 | 1.33 (1.06 to 1.67); 0.0128 | 0.38 (0.23 to 0.62); 0.0001 |
| Anxiety Sensitivity | 1.01 (0.98 to 1.03); 0.6258 | 0.97 (0.94 to 0.99); 0.0085 | 1.03 (0.99 to 1.08); 0.1377 | 0.96 (0.93 to 0.98); 0.0012 | 1.08 (1.04 to 1.12); <.0001 |
| Negative Thinking | 1 (0.98 to 1.02); 0.9791 | 1 (0.98 to 1.03); 0.831 | 0.93 (0.89 to 0.97); 0.0005 | 1.02 (1 to 1.04); 0.0697 | 0.89 (0.86 to 0.92); <.0001 |
| Impulsivity | 1.02 (0.99 to 1.05); 0.2658 | 1.02 (0.99 to 1.05); 0.2764 | 1.11 (1.05 to 1.18); 0.0002 | 1.01 (0.98 to 1.05); 0.4135 | 1.08 (1.04 to 1.13); 0.0006 |
| Sensation Seeking | 0.98 (0.96 to 1); 0.0308 | 1 (0.98 to 1.02); 0.9969 | 0.97 (0.93 to 1.01); 0.1218 | 0.99 (0.96 to 1.01); 0.2357 | 0.93 (0.9 to 0.96); <.0001 |
| SDQ Hyperactivity | 0.98 (0.94 to 1.02); 0.3368 | 1 (0.96 to 1.04); 0.985 | 0.98 (0.91 to 1.05); 0.5959 | 1 (0.96 to 1.05); 0.8402 | 0.99 (0.93 to 1.05); 0.7978 |
| SDQ Conduct | 1.09 (1.04 to 1.14); 0.0001 | 1.06 (1.01 to 1.11); 0.024 | 1.16 (1.08 to 1.25); <.0001 | 1.1 (1.05 to 1.15); <.0001 | 1.06 (0.99 to 1.13); 0.0958 |

Note: State and study did not converge (collinear with program assignment) and school type age were not different across groups in univariable models.

^1^Analyses done using PROC GLIMMIX (RSPL) in SAS Enterprise Guide 7.1

**Supplementary Table 4.** Comparing models with time as a linear and quadratic effect

S4.A Comparison of Bayesian Information Criterion estimated across five imputed datasets^1^

| Imputation # | Linear time BIC | Quadratic time BIC | Quadratic BIC – Linear BIC | Better fitting model |
| --- | --- | --- | --- | --- |
| Imputation 1 | 78051.94 | 78078.38 | 26.44 | Linear |
| Imputation 5 | 77719.24 | 77746.22 | 26.98 | Linear |
| Imputation 10 | 78363.36 | 78391.96 | 28.6 | Linear |
| Imputation 15 | 77892.14 | 77919.09 | 26.95 | Linear |
| Imputation 20 | 77842.98 | 77871.57 | 28.59 | Linear |

^1^Analyses conducted in Stata 18

S4.B Comparison of p-values for linear versus quadrative effects of time pooled across 20 imputations^1^

| Model | Low both | High Distress  RR (95% CI); p-value | High Alcohol  RR (95% CI); p-value | Distress + Alcohol  RR (95% CI); p-value |
| --- | --- | --- | --- | --- |
| Linear Model | | | | |
| Linear time | *reference* | 1 (1 to 1.01); 0.013 | 1.1 (1.09 to 1.11); <0.001 | 1.09 (1.08 to 1.1); <0.001 |
| Quadratic model | | | | |
| Linear time | *reference* | 1 (0.99 to 1.01); 0.897 | 1.09 (1.07 to 1.12); <0.001 | 1.09 (1.06 to 1.11); <0.001 |
| Quadratic time | *reference* | 1 (1 to 1); 0.361 | 1 (1 to 1); 0.399 | 1 (1 to 1); 0.708 |

^1^Analyses conducted in Stata 18

**Supplementary Table 5**. Main model with Q4 as the reference group

|  | Basic Model  Relative Risk Ratio (95% Confidence Interval); p-value | | | Adjusted Model  Relative Risk Ratio (95% Confidence Interval); p-value | | |
| --- | --- | --- | --- | --- | --- | --- |
| Reference = High Alcohol + Distress | Low both | Distress only | Alcohol only | Low both | Distress only | Alcohol only |
| Female sex (ref=male) | 0.52 (0.4 to 0.69); 0 | 1.21 (0.95 to 1.53); 0.127 | 0.45 (0.31 to 0.65); 0 | 0.5 (0.4 to 0.62); 0 | 1.02 (0.81 to 1.28); 0.868 | 0.52 (0.37 to 0.75); 0 |
| CAP Study (ref=CSC) | 0.33 (0.21 to 0.51); 0 | 0.45 (0.29 to 0.69); 0 | 1.07 (0.59 to 1.94); 0.832 | 0.39 (0.27 to 0.58); 0 | 0.45 (0.3 to 0.67); 0 | 1.08 (0.63 to 1.85); 0.779 |
| Time (1 month) | 0.92 (0.9 to 0.93); 0 | 0.93 (0.92 to 0.94); 0 | 1.04 (1.02 to 1.06); 0 | 0.91 (0.9 to 0.93); 0 | 0.93 (0.92 to 0.94); 0 | 1.03 (1.01 to 1.05); 0.001 |
| Climate SU (ref=Control) | 0.45 (0.25 to 0.83); 0.01 | 0.6 (0.35 to 1.02); 0.061 | 0.99 (0.5 to 1.97); 0.978 | 0.56 (0.36 to 0.89); 0.014 | 0.6 (0.37 to 0.98); 0.041 | 1.07 (0.56 to 2.05); 0.83 |
| Climate MH (ref=Control) | 0.42 (0.21 to 0.84); 0.014 | 0.52 (0.31 to 0.87); 0.013 | 0.93 (0.45 to 1.96); 0.855 | 0.52 (0.29 to 0.91); 0.023 | 0.55 (0.33 to 0.9); 0.018 | 0.94 (0.47 to 1.9); 0.872 |
| PreVenture (ref=Control) | 0.32 (0.16 to 0.65); 0.002 | 0.41 (0.25 to 0.68); 0.001 | 1.13 (0.43 to 2.96); 0.808 | 0.43 (0.23 to 0.81); 0.009 | 0.47 (0.28 to 0.81); 0.006 | 1.09 (0.45 to 2.62); 0.849 |
| CSC (ref=Control) | 0.35 (0.15 to 0.8); 0.013 | 0.43 (0.2 to 0.93); 0.032 | 1.16 (0.58 to 2.33); 0.672 | 0.49 (0.25 to 0.94); 0.031 | 0.45 (0.22 to 0.93); 0.032 | 1.28 (0.68 to 2.44); 0.444 |
| CAP (ref=Control) | 0.73 (0.39 to 1.39); 0.34 | 0.55 (0.31 to 0.97); 0.039 | 1.93 (0.71 to 5.23); 0.197 | 0.67 (0.41 to 1.12); 0.129 | 0.6 (0.37 to 0.99); 0.045 | 1.74 (0.72 to 4.2); 0.216 |
| Climate SU x month | 1.01 (1 to 1.03); 0.137 | **1.02 (1 to 1.03); 0.041** | 0.99 (0.97 to 1.01); 0.221 | 1.01 (1 to 1.03); 0.129 | **1.02 (1 to 1.03); 0.033** | 0.99 (0.97 to 1.01); 0.266 |
| Climate MH x month | 1.01 (0.99 to 1.03); 0.257 | 1.01 (0.99 to 1.03); 0.242 | 1.01 (0.98 to 1.03); 0.608 | 1.01 (0.99 to 1.03); 0.269 | 1.01 (0.99 to 1.03); 0.241 | 1.01 (0.98 to 1.03); 0.557 |
| PreVenture x month | **1.04 (1.02 to 1.06); 0.001** | **1.02 (1 to 1.04); 0.018** | 0.99 (0.97 to 1.02); 0.488 | **1.04 (1.01 to 1.06); 0.001** | **1.02 (1 to 1.04); 0.019** | 0.99 (0.97 to 1.02); 0.648 |
| CSC x month | **1.03 (1.01 to 1.05); 0.003** | 1.02 (1 to 1.04); 0.073 | 0.98 (0.96 to 1.01); 0.145 | **1.03 (1.01 to 1.05); 0.004** | 1.02 (1 to 1.04); 0.077 | 0.98 (0.96 to 1.01); 0.189 |
| CAP x month | 1.02 (0.99 to 1.04); 0.187 | **1.02 (1.01 to 1.04); 0.006** | 0.98 (0.96 to 1); 0.098 | 1.02 (0.99 to 1.04); 0.218 | **1.02 (1.01 to 1.04); 0.009** | 0.98 (0.96 to 1); 0.113 |
| Other English-Speaking Country |  |  |  | 0.68 (0.54 to 0.85); 0.001 | 0.77 (0.59 to 1.01); 0.058 | 0.79 (0.59 to 1.06); 0.115 |
| Other Non-English-Speaking Country |  |  |  | 2.17 (1.57 to 3.01); 0 | 2.82 (2.07 to 3.83); 0 | 0.68 (0.47 to 0.98); 0.037 |
| SDQ Hyperactivity |  |  |  | 0.76 (0.73 to 0.79); 0 | 0.96 (0.92 to 1); 0.042 | 0.82 (0.78 to 0.86); 0 |
| SDQ Conduct |  |  |  | 0.85 (0.82 to 0.89); 0 | 0.93 (0.89 to 0.97); 0.002 | 0.94 (0.89 to 0.99); 0.027 |
| Anxiety Sensitivity |  |  |  | 0.96 (0.93 to 0.98); 0 | 1.08 (1.05 to 1.11); 0 | 0.9 (0.88 to 0.93); 0 |
| Impulsivity |  |  |  | 0.97 (0.94 to 1.01); 0.13 | 1.01 (0.98 to 1.04); 0.553 | 1.06 (1.01 to 1.1); 0.01 |
| Sensation Seeking |  |  |  | 0.87 (0.85 to 0.89); 0 | 0.89 (0.87 to 0.91); 0 | 1.03 (0.99 to 1.06); 0.137 |
| Negative Thinking |  |  |  | 0.78 (0.77 to 0.8); 0 | 0.98 (0.95 to 1); 0.036 | 0.91 (0.88 to 0.94); 0 |

**Supplementary Table 6**. Basic model sensitivity analyses for missing data: complete case and inverse probability weights

|  | Complete Case Model  Relative Risk Ratio (95% Confidence Interval); p-value | | | Model with Inverse Probability Weights for missing*  Relative Risk Ratio (95% Confidence Interval); p-value | | |
| --- | --- | --- | --- | --- | --- | --- |
| Reference = Low in both | Distress only | Alcohol only | Distress + Alcohol | Low both | Distress only | Alcohol only |
| Female sex (ref=male) | 2.14 (1.82 to 2.52); 0 | 0.74 (0.49 to 1.12); 0.156 | 2.1 (1.51 to 2.93); 0 | 2.17 (1.83 to 2.57); 0 | 0.73 (0.47 to 1.15); 0.178 | 2.08 (1.46 to 2.97); 0 |
| CAP Study (ref=CSC) | 1.19 (0.89 to 1.59); 0.243 | 3.11 (1.57 to 6.16); 0.001 | 4.26 (2.58 to 7.04); 0 | 1.06 (0.74 to 1.51); 0.758 | 3.82 (1.81 to 8.03); 0 | 4.36 (2.6 to 7.31); 0 |
| Time (1 month) | 1.01 (1 to 1.01); 0.189 | 1.13 (1.11 to 1.16); 0 | 1.11 (1.09 to 1.13); 0 | 1 (0.99 to 1.01); 0.635 | 1.13 (1.11 to 1.16); 0 | 1.11 (1.09 to 1.13); 0 |
| Climate SU (ref=Control) | 1.22 (0.91 to 1.65); 0.184 | 2.17 (0.93 to 5.08); 0.074 | 2.5 (1.17 to 5.35); 0.018 | 1.18 (0.85 to 1.63); 0.316 | 2.2 (0.88 to 5.47); 0.091 | 2.4 (1.04 to 5.54); 0.041 |
| Climate MH (ref=Control) | 1.17 (0.82 to 1.67); 0.401 | 2.22 (0.82 to 6.02); 0.119 | 3.07 (1.24 to 7.6); 0.015 | 1.06 (0.75 to 1.5); 0.75 | 2.29 (0.85 to 6.17); 0.101 | 3.1 (1.32 to 7.27); 0.009 |
| PreVenture (ref=Control) | 1.25 (0.86 to 1.82); 0.247 | 3.03 (1.1 to 8.32); 0.032 | 4 (1.68 to 9.52); 0.002 | 1.35 (0.87 to 2.08); 0.179 | 3.69 (1.19 to 11.44); 0.024 | 3.6 (1.36 to 9.56); 0.01 |
| CSC (ref=Control) | 1.13 (0.78 to 1.66); 0.513 | 3.38 (1.27 to 8.99); 0.014 | 3.82 (1.24 to 11.76); 0.02 | 1.06 (0.71 to 1.58); 0.768 | 3.96 (1.45 to 10.83); 0.007 | 3.62 (1.1 to 11.9); 0.034 |
| CAP (ref=Control) | 0.76 (0.43 to 1.34); 0.341 | 2.58 (1.04 to 6.38); 0.04 | 1.54 (0.69 to 3.42); 0.294 | 0.73 (0.39 to 1.36); 0.322 | 2.39 (0.9 to 6.39); 0.082 | 1.46 (0.61 to 3.49); 0.396 |
| Climate SU x month | 1 (0.99 to 1.02); 0.453 | **0.97 (0.95 to 1); 0.032** | 0.99 (0.96 to 1.01); 0.17 | 1 (0.99 to 1.01); 0.653 | **0.97 (0.95 to 1); 0.037** | 0.99 (0.96 to 1.01); 0.257 |
| Climate MH x month | 1 (0.99 to 1.01); 0.727 | 0.99 (0.96 to 1.02); 0.656 | 0.99 (0.96 to 1.01); 0.265 | 1 (0.99 to 1.01); 0.835 | 0.99 (0.96 to 1.02); 0.603 | 0.98 (0.95 to 1); 0.112 |
| PreVenture x month | **0.98 (0.97 to 1); 0.047** | **0.96 (0.92 to 0.99); 0.013** | **0.95 (0.93 to 0.98); 0.001** | 0.99 (0.98 to 1); 0.074 | **0.95 (0.92 to 0.98); 0.004** | **0.96 (0.94 to 0.99); 0.002** |
| CSC x month | 0.99 (0.98 to 1); 0.134 | **0.95 (0.93 to 0.98); 0** | **0.97 (0.94 to 1); 0.032** | 0.99 (0.98 to 1); 0.187 | **0.95 (0.92 to 0.97); 0** | **0.97 (0.94 to 1); 0.038** |
| CAP x month | 1.01 (0.99 to 1.02); 0.399 | **0.96 (0.93 to 1); 0.026** | 0.97 (0.94 to 1.01); 0.147 | 1.01 (0.99 to 1.03); 0.328 | **0.96 (0.93 to 1); 0.035** | 0.98 (0.94 to 1.01); 0.201 |

**IPW was done in long format with stabilized weights. Predicted probabilities for observed 4-quadrant outcomes were generated using logistic regression with study, sex, country of birth, ADHD symptoms, conduct symptoms, anxiety sensitivity, impulsivity, sensation seeking, and negative thinking as predictors (informed by previous missing data explorations).*

**Supplementary Table 7**. Main analysis with categorical time. Please note, that statistical power is lost when categorizing continuous variables – in this case, time. Additionally, 30 and 36 month outcomes had to be combined as CAP and CSC had different final follow-up time points and several time points are not included in this analysis (e.g., 6 month follow-ups).

|  | Basic Model  Relative Risk Ratio (95% Confidence Interval); p-value | | |
| --- | --- | --- | --- |
| Reference = No Alcohol + Low/No Distress | Distress only | Alcohol only | Distress + Alcohol |
| Female sex (ref=male) | 2.03 (1.71 to 2.41); 0 | 0.77 (0.51 to 1.15); 0.197 | 2.05 (1.49 to 2.81); 0 |
| CAP Study (ref=CSC) | 1.29 (0.96 to 1.73); 0.09 | 3.49 (1.74 to 6.97); 0 | 4.86 (2.91 to 8.1); 0 |
| 12 months | 1.18 (0.94 to 1.48); 0.165 | 3.83 (1.87 to 7.85); 0 | 2.63 (1.51 to 4.59); 0.001 |
| 24 months | 1.07 (0.85 to 1.34); 0.578 | 15.57 (8.13 to 29.82); 0 | 10.05 (6.06 to 16.66); 0 |
| 30/36 months | 1.23 (0.98 to 1.56); 0.077 | 39.8 (21.59 to 73.36); 0 | 19.22 (10.86 to 34.03); 0 |
| Climate SU (ref=Control) | 1.28 (0.88 to 1.86); 0.201 | 2.19 (0.9 to 5.33); 0.083 | 2.12 (0.98 to 4.59); 0.057 |
| Climate MH (ref=Control) | 1.25 (0.81 to 1.91); 0.315 | 2.4 (0.95 to 6.1); 0.065 | 2.09 (0.95 to 4.59); 0.065 |
| PreVenture (ref=Control) | 1.12 (0.74 to 1.72); 0.588 | 3.64 (1.26 to 10.56); 0.017 | 2.56 (1.1 to 5.96); 0.029 |
| CSC (ref=Control) | 1.19 (0.76 to 1.87); 0.444 | 3.1 (1.16 to 8.28); 0.024 | 3.08 (1.08 to 8.79); 0.035 |
| CAP (ref=Control) | 0.82 (0.42 to 1.62); 0.573 | 3.05 (1.1 to 8.45); 0.032 | 1.13 (0.55 to 2.3); 0.738 |
| 12 months x Climate SU | 0.94 (0.65 to 1.35); 0.735 | 0.64 (0.28 to 1.46); 0.293 | 1.03 (0.53 to 1.99); 0.929 |
| 12 months x Climate MH | 0.77 (0.56 to 1.07); 0.121 | 0.79 (0.34 to 1.84); 0.589 | 1.79 (0.86 to 3.74); 0.119 |
| 12 months x PreVenture | 0.73 (0.45 to 1.18); 0.195 | **0.34 (0.15 to 0.79); 0.012** | 0.6 (0.25 to 1.44); 0.247 |
| 12 months x CSC | 0.72 (0.49 to 1.07); 0.105 | 0.66 (0.31 to 1.4); 0.277 | 0.74 (0.37 to 1.49); 0.401 |
| 12 months x CAP | 0.82 (0.5 to 1.35); 0.429 | **0.3 (0.13 to 0.7); 0.005** | 0.82 (0.43 to 1.57); 0.543 |
| 24 months x Climate SU | 1.24 (0.87 to 1.76); 0.233 | 0.54 (0.25 to 1.17); 0.118 | 0.79 (0.42 to 1.46); 0.446 |
| 24 months x Climate MH | 0.87 (0.62 to 1.22); 0.418 | 0.86 (0.4 to 1.85); 0.693 | 0.82 (0.41 to 1.65); 0.574 |
| 24 months x PreVenture | 0.83 (0.51 to 1.33); 0.433 | **0.26 (0.12 to 0.58); 0.001** | **0.43 (0.19 to 0.96); 0.039** |
| 24 months x CSC | 0.86 (0.6 to 1.23); 0.421 | **0.45 (0.21 to 0.95); 0.035** | 0.61 (0.32 to 1.16); 0.13 |
| 24 months x CAP | 1.24 (0.71 to 2.16); 0.455 | **0.44 (0.2 to 0.98); 0.045** | 0.65 (0.34 to 1.25); 0.196 |
| 30/36 months x Climate SU | 0.98 (0.69 to 1.38); 0.9 | **0.42 (0.2 to 0.86); 0.018** | 0.81 (0.42 to 1.57); 0.542 |
| 30/36 months x Climate MH | 1.02 (0.73 to 1.44); 0.906 | 0.68 (0.32 to 1.48); 0.331 | 0.93 (0.44 to 1.96); 0.846 |
| 30/36 months x PreVenture | 0.6 (0.34 to 1.08); 0.091 | **0.29 (0.1 to 0.82); 0.02** | 0.43 (0.17 to 1.07); 0.071 |
| 30/36 months x CSC | 0.78 (0.55 to 1.1); 0.159 | **0.22 (0.11 to 0.44); 0** | **0.46 (0.22 to 0.96); 0.038** |
| 30/36 months x CAP | 1.03 (0.55 to 1.91); 0.931 | **0.33 (0.13 to 0.85); 0.021** | 0.82 (0.34 to 1.97); 0.654 |

**Supplementary Table 8**. Differential program effects by sex^1^

| Model | High Distress  RR (95% CI); p-value | High Alcohol  RR (95% CI); p-value | Distress + Alcohol  RR (95% CI); p-value |
| --- | --- | --- | --- |
| Female x month x Climate SU | 0.97 (0.46 to 2.04); 0.936 | 0.58 (0.17 to 1.99); 0.386 | 0.54 (0.16 to 1.84); 0.321 |
| Female x month x Climate MH | 0.9 (0.37 to 2.2); 0.808 | 0.85 (0.24 to 3.04); 0.807 | 1.13 (0.26 to 4.89); 0.87 |
| Female x month x PreVenture | 1.59 (0.43 to 5.84); 0.482 | 0.43 (0.07 to 2.52); 0.344 | 0.4 (0.09 to 1.87); 0.241 |
| Female x month x CSC | 0.97 (0.45 to 2.07); 0.938 | 1.19 (0.32 to 4.4); 0.793 | 0.73 (0.2 to 2.7); 0.632 |
| Female x month x CAP | 1.68 (0.54 to 5.2); 0.368 | 0.99 (0.22 to 4.45); 0.989 | 0.75 (0.13 to 4.31); 0.74 |

Note: Multilevel mixture models pooled across imputations 1-10 and adjusted for study, sex, time, program, sex x time, time x program

^1^Analyses conducted in Stata 18

**Supplementary Materials Figure 2.** Model-predicted probabilities (Imputation #20, Model 1)

**Supplementary Table 9.A** Predicted probabilities of Quadrant 1 by group across time (imp 20)

|  | Baseline | 12 months | 24 months | 36 months |
| --- | --- | --- | --- | --- |
| Control | 70.6 (67.2-74) | 64.2 (60.8-67.6) | 53.7 (49.7-57.8) | 40.3 (35.5-45.1) |
| Climate SU | 66.1 (63.8-68.4) | 58.9 (55.6-62.1) | 49 (44.2-53.9) | 37.9 (32-43.8) |
| Climate MH | 66.2 (61.8-70.6) | 58.2 (53.3-63.1) | 46.7 (41.8-51.7) | 33.7 (29.1-38.3) |
| PreVenture | 63.5 (58.6-68.4) | 59.8 (54.5-65) | 53.8 (47.9-59.7) | 46.1 (39.4-52.8) |
| CSC | 64 (59.1-69) | 58.6 (53.1-64.2) | 51.3 (45-57.7) | 42.9 (36-49.8) |
| CAP | 71.4 (66.6-76.3) | 64.5 (60.8-68.1) | 55 (49.7-60.4) | 43.9 (35.6-52.3) |

**Supplementary Table 9.B** Predicted probabilities of Quadrant 2 by group across time (imp 20)

|  | Baseline | 12 months | 24 months | 36 months |
| --- | --- | --- | --- | --- |
| Control | 23.4 (20.7-26) | 22.8 (20.8-24.9) | 21 (19.2-22.8) | 17.6 (15.6-19.7) |
| Climate SU | 24.9 (22.5-27.4) | 24.7 (22.4-27.1) | 23.4 (20.9-25.9) | 20.8 (17.9-23.6) |
| Climate MH | 24 (21.3-26.7) | 22.9 (20.4-25.4) | 20.3 (17.5-23.1) | 16.5 (13.3-19.7) |
| PreVenture | 25.3 (22.1-28.5) | 22.2 (19.1-25.3) | 18.7 (15.7-21.7) | 15 (12.2-17.9) |
| CSC | 23.8 (20.2-27.4) | 22 (18.9-25.1) | 19.7 (16.7-22.6) | 16.8 (13.8-19.9) |
| CAP | 19.7 (14.2-25.1) | 19.7 (15.3-24.2) | 19 (15.2-22.8) | 17.3 (14-20.7) |

**Supplementary Table 9.C** Predicted probabilities of Quadrant 3 by group across time (imp 20)

|  | Baseline | 12 months | 24 months | 36 months |
| --- | --- | --- | --- | --- |
| Control | 2.8 (1.7-4) | 6.9 (4.8-9.1) | 14.6 (11-18.2) | 25.4 (20.1-30.8) |
| Climate SU | 4.2 (2.7-5.7) | 8.1 (5.3-11) | 14.2 (9.4-19) | 21.9 (15-28.8) |
| Climate MH | 4.2 (2.2-6.2) | 9.7 (6.3-13.1) | 18.9 (14.3-23.6) | 31.1 (25.2-37) |
| PreVenture | 5.3 (3.1-7.6) | 9.2 (5.8-12.6) | 14.7 (9.8-19.6) | 21.7 (15.3-28.1) |
| CSC | 5.9 (3.5-8.3) | 9.8 (6-13.7) | 15.2 (9.7-20.8) | 21.8 (14.2-29.4) |
| CAP | 5.4 (3.5-7.3) | 9.8 (6-13.6) | 16.4 (9.9-22.8) | 24.5 (15.3-33.7) |

**Supplementary Table 9.D** Predicted probabilities of Quadrant 3 by group across time (imp 20)

|  | Baseline | 12 months | 24 months | 36 months |
| --- | --- | --- | --- | --- |
| Control | 3.2 (2.4-4) | 6.1 (5-7.1) | 10.7 (9.1-12.3) | 16.6 (14.3-19) |
| Climate SU | 4.8 (3.6-5.9) | 8.3 (6.5-10) | 13.4 (10.9-15.9) | 19.4 (16.4-22.3) |
| Climate MH | 5.6 (3.8-7.3) | 9.3 (7.1-11.4) | 14 (12-16.1) | 18.7 (16.7-20.8) |
| PreVenture | 5.8 (3.8-7.9) | 8.9 (6.3-11.4) | 12.8 (9.6-15.9) | 17.2 (13.3-21.1) |
| CSC | 6.2 (3.6-8.9) | 9.5 (6.5-12.5) | 13.8 (10.8-16.7) | 18.5 (15.9-21.1) |
| CAP | 3.5 (2.4-4.7) | 6 (4.5-7.4) | 9.6 (7.4-11.8) | 14.2 (10.9-17.6) |
